# Preparation of Pulp-Cavity and Canals

**Published:** 1896-06

**Authors:** J. Foster Flagg

**Affiliations:** Philadelphia.


ARTICLE IV.

### PREPARATION OF PULP-CAVITY AND CANALS.

DR. J. FOSTER FLAGG, PHILADELPHIA.

Pulp-cavity work necessitates the entrance into the pulp-cavity, and this must be affected through at least a partial devitalization of the pulp; if this has been the outcome of other than medicinal application, the methods of entrance are various from only two practical considerations: First, removal of filling, naturally presupposing such to exist, and entering pulp-cavity from cavity of decay, recognizing difficulties of canal work and endeavoring to overcome them; and second, ignoring all previous considerations, and operating with the recognition only of the pulp-cavity and canal requirements, making such entrance as shall eventuate in a "tap," with all its advantages both for present and future possible complications.

But if, on the contrary, such pulp-irritation exists as to demand its devitalization, there is probably no other application as yet more universally resorted to than some combination of arsenic.

It is in connection with these that I have long combated one consideration which, from constant mention and frequent discussion, I hold to be yet almost universally regarded as of much importance, and this is the length of duration of the application.

Twenty-four to forty eight hours, and by some even a week, are spoken of as though they were alike productive of good and preventive of undesirable results.

For good results I have, for more than thirty years, advocated the continuance of arsenical applications till the desired result was accomplished regardless of any five hours or five days or five weeks or five months (if circumstances should require it), in the full faith that length of duration of application was of no importance and that in fully-formed teeth (and I presume no one would ever risk making an arsenical application in any others) no undesirable sequences would ever follow.

As proof of this I had, in years long gone by, ample opportunity for following the frequent applications of arsenic which in those days were made for the obtunding of sensitive dentine, and which, having devitalized the pulps, had been the means of quieting all trouble till, in process of time, these pulps had putresced and had induced peridental irritation from evolution of mephitic gas, precisely as they would have done had they been devitaliz-in any other way.

Following such hintings I was obliged to abandon all time considerations as associated with assenical applications, and have thus been enabled to make all pulp-cavity work, dependent on these, in complete consonance with my patients' or my own convenience.

In this wise I always prefer that weeks rather than hours shall be the length of duration of applications; and so it is that when serious pulp-trouble unfortunately antecedes arranged summerings or other long absences from home, applications can be made which will effectually preclude any probability of toothache during such times and which will, most probably, bring the tooth, on return of patient, in excellent condition for thorough and painless treatment.

I urge the need for proper placing of the arsenic, the precluding of pressure, the maintenance of position, and the prevention of leakage.

With these views it becomes not only undesirable, but culpable, that wax, cotton with sandarac or other gum varnishes, or even gutta-percha or any other than the three coverings mentioned should be used, as no others permit the secure and proper utilization of arsenical applications.

Supposing complete devitalization has been effected pulp-cavity work becomes simple and usually easy, as nothing is indicated except the obliteration of the walls of the pulp-cavity; by this we have the mouths of the canals defined and the bulbous portion of the pulp removed, and now the trouble begins.

It is regarded, I believe, as of first importance that all the pulp-structure should be removed, and to this end scores of pages in our journals and days of discussion in our societies are given to the methods and instruments for the effecting of this purpose, all having in view the following of every canal to its apical end, and some asserting positively that they always attain this "all-important" result.

I not infrequently meet with canals whose entire length I feel it equally imprudent and impossible to explore.

More than this, I am cognizant of roots, the canals of which I know cannot be followed with the teeth in the hand. How much less, then, would this be possible in the mouth? In fact, this is just the barrier which prevents belief in any statement beyond respectable ability as associated with canal work.

Therefore, as in a large proportion of canals, it is comparatively easy to go to the very end, I regard, as of first importance, that the apical foramen shall not be passed, and, above all, not enlarged.

That it should not be enlarged is because just in proportion as this foramen is normally small—even at its smallest—so is it possible to make a good canal filling whether that filling be of gold or of inspissated or even merely fluid medicament; and, inversely, every enlargement of this orifice, however trifling, in equally just proportion renders it less possible to make a perfect filling of long continuance.

I shall ignore the whole question of medicating through an enlarged foramen, as such practice could only be indulged in abscess without fistula, and, in my opinion, even then would be bad practice.

My experience has taught me that perforation of the root is most frequently a result of attempts at enlarging and following canals in the determined work of removing every particle of pulp-tissue; and I would ask which is preferable, that a portion of pulp-tissue remain or that a root be unperforated?

A portion of drill should not be broken off and left in a canal. Why should these broken drills be gotten out? This is a long story which must be shortly told. It is not that of themselves they would do harm, but that they obstruct an open canal.

Who has not experienced the giving of almost immediate relief to teeth so tender that it hurt even to look at them, and that seemed a foot, aye, three feet, longer than any other, so soon as the last portion of obstruction was removed from the canal ? and how almost utterly ineffectual are efforts, frequently, till this has been accomplished ?

Just here is the battle-ground between those who believe that if every particle of pulp-tissue is removed and the canal is filled with an impervious and indestructable material, no further trouble will ever come, and, as it is expressed, "the work is thoroughly done and will last for life," and those who have no faith in such belief, and who, on the contrary, hold it as much more thorough to try and fix things so that they will remain fixed, if, fortunately, no trouble comes, but that there will also be ample preparation for future relief in case exigencies demand it.

But I should remove every possible portion of pulp.

No one will deny that just in proportion as this is done, so it is reasonable to expect success. Yet, there are plenty of cases of but very partial removal of pulp which have given successes of remarkable duration.

And I also hold that because of the possibility of doing much with antiseptics to preclude any likelihood of putrescence of remaining portions of pulp, and of the ease with which arrangements for venting and future purifying of pulp-cavity and canals can be made, it is equally unwarrantable to take any risk which could, by any possibility, eventuate in irremediable injury, for the purpose of accomplishing so comparatively unimportant a result as is absolute pulp-extirpation.

Viewing this work of canal entrance and cleansing as very important, I desire to refer to the suggestion of Dr. J. R. Callahan, as presented in his paper of July, 1894, in which he advocated the opening of canals by means of "50 per cent aqueous solution of sulfuric acid."

After a year's experience of this method I can speak of it in terms of unqualified approval, and say that I regard it as the step in advance, in connection with canal work, which has been given us during the past twenty-five years.

Nor only in fine, tortuous, and ordinarily inaccessible canals does this process make easy much that would be both difficult and dangerous, but it affords the most prompt, the most efficacious, and the most satisfactory method of entering, cleansing, and beautifully preparing the largest and most accessible canals.

I would call attention to his "pumping motion" of using the probes, both small and great, as a decided improvement over the dangerous rotation and twisting motion by which alone drills or broaches can be made available, for by this modification the danger of breaking probes in canals is almost entirely abrogated.

I have found that there is yet danger of this if steel probes are used, as these in time become coroded by the dilute acid. I also find that electro-plated probes soon lose their gold, but with iridium-platinum and platinum-gold wire probes I have had comfort in doing and demonstrating this king of work, for which I would tender Dr Callahan hearty thanks.—*Cosmos.*

